# Healthy beverage index could decrease odds of metabolic syndrome: A cross‐sectional study

**DOI:** 10.1002/fsn3.3415

**Published:** 2023-06-20

**Authors:** Mehran Nouri, Zainab Shateri, Mahshid Rezaei, Ali Zangene, Reza Homayounfar, Parisa Keshani

**Affiliations:** ^1^ Department of Community Nutrition, School of Nutrition and Food Sciences Shiraz University of Medical Sciences Shiraz Iran; ^2^ Student Research Committee Shiraz University of Medical Sciences Shiraz Iran; ^3^ Health Policy Research Center, Institute of Health Shiraz University of Medical Sciences Shiraz Iran; ^4^ Student Research Committee Ahvaz Jundishapur University of Medical Sciences Ahvaz Iran; ^5^ Science and Research Branch Islamic Azad University of Tehran Tehran Iran; ^6^ Department of Community Nutrition, School of Nutrition and Food Sciences Isfahan University of Medical Sciences Isfahan Iran; ^7^ Non‐Communicable Diseases Research Center Fasa University of Medical Sciences Fasa Iran

**Keywords:** blood pressure, fasting blood glucose, healthy beverage index, lipid profile, metabolic syndrome, waist circumference

## Abstract

Some studies indicated that sugar‐sweetened beverages (SSBs) were related to MetS risk, and others found no relationship between MetS and SSBs. Therefore, the present study aimed to investigate the relationship between healthy beverage index (HBI) and MetS in Iranian adults. This cross‐sectional study was performed on baseline data FASA cohort. Out of 10,127 participants in the FASA cohort study, 8838 participants were included in this study. The National Cholesterol Education Program's Adult Treatment Panel (ATP) III was used for MetS definition. The HBI was calculated by a 125‐item food frequency questionnaire with standard criteria. The association between HBI and MetS and its components was evaluated by univariate regression. Multivariate regression with the backward method was used for adjusting confounders. *p* < .05 was considered as statistically significant. Compared to the first quartile, it was observed that HBI in the last quartile was significantly related to lower odds of MetS in the multivariate analysis (odds ratio [OR] = 0.72; 95% confidence interval [CI]: 0.60–0.87, *p* < .001). Also, we observed a significant association between the last quartile of HBI with lower odds of high waist circumference (WC) (OR = 0.55; 95% CI: 0.45–0.67, *p* < .002). Our findings showed that the higher HBI score reduced MetS odds and WC. Therefore, to reduce the odds of MetS, a healthy pattern of beverage consumption, including drinking water, low‐fat milk, unsweetened tea, and coffee, and reducing the consumption of SSB are recommended. More studies are needed to confirm the findings.

## Graphical abstract

Our findings showed that the higher HBI score reduced MetS risk and WC. Therefore, to prevent MetS, it is recommended to have a good pattern of beverage consumption, such as drinking more water, low‐fat milk, unsweetened tea, and coffee, and reducing the consumption of SSB. More studies are needed to confirm the findings.

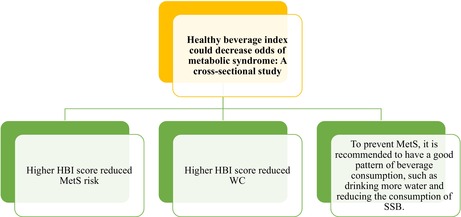

## 1 INTRODUCTION

Metabolic syndrome (MetS) is a major and growing clinical and public health challenge in the world due to urbanization, excess energy intake, sedentary life habits, and increasing obesity (Kaur, 2014). This syndrome is defined by clinical, biochemical, physiological, and metabolic factors characterized by hypertension, insulin resistance, central obesity, dyslipidemia, and glucose intolerance (Wittcopp & Conroy, 2016). These risk factors can increase the prevalence of cardiovascular diseases (CVDs) and type 2 diabetes mellitus (Wang et al., 2020). The prevalence of MetS varies depending on the definition used, age, gender, ethnic background, and socioeconomic status of the study groups (Wang et al., 2020). According to a study, this syndrome's overall prevalence in Iran is 32.6% (Farmanfarma et al., 2019).

Dietary habits are related to some factors included in the definition of MetS and can play an important role in preventing obesity and MetS (Djousse et al., 2010). Liquids and beverages are an important part of each individual's diet, and about 80% of each person's fluid intake comes from beverages (EFSA Panel on Dietetic Products & Allergies, 2010). Adequate fluid intake has several health benefits, especially some like tea, coffee, and milk is important for maintaining blood glucose homeostasis (Clark et al., 2013) and the prevention of some diseases such as diabetes mellitus (Poole et al., 2017) and CVDs (Ding et al., 2014). On the other hand, consuming a high amount of sugar‐sweetened beverages (SSBs) may cause overweight/obesity (Malik & Hu, 2022; Twarog et al., 2020), increases inflammatory markers (Lin et al., 2020), impaired lipid metabolism (Aeberli et al., 2011), and be related to adverse levels of multiple cardiometabolic biomarkers (Yu et al., 2018). Reducing SSB intake is associated with a significant reduction in blood pressure and the risk of CVDs (Brown et al., 2011; Johnson et al., 2009). However, few studies have examined the overall quality of daily beverage intake as a pattern (Duffey & Poti, 2016; Kawada, 2016). Therefore, the healthy beverage index (HBI) was proposed as a general concept to assess the overall quality of beverage consumption and its association with health in epidemiological studies of nutrition (Duffey & Davy, 2015).

There are contradictory results regarding the association of MetS with HBI scores. Some studies indicated that beverage intake was related to MetS risk (Malik et al., 2010; Sturt, 2011), and others found no relationship between MetS and beverage intake (Khosravi‐Boroujeni et al., 2012; Trapp et al., 2020). Therefore, the present study aimed to investigate the relationship between HBI and MetS in Iranian adults.

## 2 METHODS

### 2.1 Study population

**FIGURE 1:**
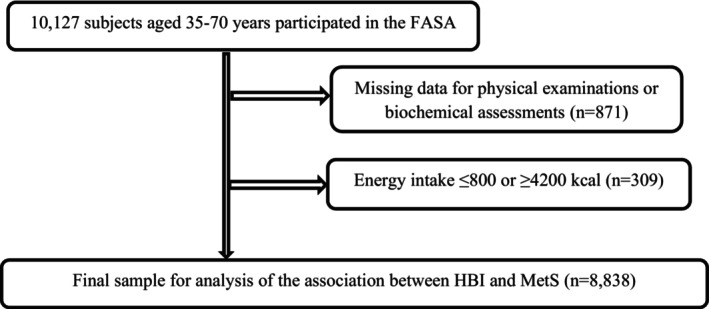
Study flow diagram. HBI, healthy beverage index; MetS, metabolic syndrome; SSB, sugar‐sweetened beverage.

This cross‐sectional study has been conducted to assess the noncommunicable disease (NCD) risk factors in the rural population of FASA (Fars province of Iran) who participated in the Prospective Epidemiological Research Studies in Iran (PERSIAN) FASA cohort. At first, 11,079 adults were invited from Sheshdeh and 24 nearby city villages. General, medical and nutritional information, physical assessments, and biological sampling for laboratory examination were collected by educated experts at the beginning of the study (the details of this study have been published previously). A semi‐125 food frequency questionnaire (FFQ) was used for food intake evaluation (Farjam et al., 2016). Daily caloric intake ≤800 or ≥4200 kcal/day was excluded to avoid dietary misclassification. Figure 1 shows the study participants' selection. This study was approved by the Medical Research and Ethics Committee of Shiraz University of Medical Science (IR.SUMS.REC.1401.363).

### 2.2 Data collection

Physical examinations were done by physicians. Bioelectrical impedance analysis (Tanita BC‐418; Tanita Corp.) was used to measure height and weight. Also, the nonstretchable tape was used to evaluate hip circumference (HC) and waist circumference (WC). Systolic blood pressure (SBP) and diastolic blood pressure (DBP) were checked in both arms after 5 min in a sitting position and repeated after 15 min. Also, based on the study protocol, after 12‐ to 14‐h fasting, a 25‐mL sample was taken from all subjects, and samples were stored at −80°C for further analysis. Lipid profiles and fasting blood sugar (FBS) were assessed by frozen plasma samples (Farjam et al., 2016).

After breakfast, the participants filled out the study questionnaire by interview. This questionnaire included general, nutritional, and medical questions. General information was divided into individual, socioeconomic, lifestyle, home situation, occupation, and anthropometric data. Medical data included NCDs' history, physical activity, and smoking. For the evaluation of nutritional intake and habits in the last year, a semi‐FFQ was used (Farjam et al., 2016). Then, all food items were altered to grams, and then energy, macro‐, and micronutrient intake were measured by the Nutritionist IV software (version 7.0; N‐Squared Computing).

### 2.3 HBI score

Based on Duffey and Davy's method, we calculated the HBI score (Duffey & Davy, 2015). Total beverages were subdivided into 10 strata: water (0–15 points), low‐fat milk (<1.5% fat or fat free) (0–5 points), full‐fat milk (˃1.5% fat) (0–5 points), tea and unsweetened coffee (0–5 points), diet drinks (artificially sweetened beverages and noncalorically) (0–5 points), natural fruit juice (0–5 points), alcohol (beer, liquor, and wine) (0–5 points), SSBs (soda and sweetened coffee) (0–15 points), total beverage energy (0–20 points), and met fluid requirement (0–20 points). Total HBI ranges from 0 to 100, and a higher score shows better adherence to a healthier HBI pattern. In this study, alcohol content and diet drink were not reported. Therefore, the maximum HBI score was 90 (Jalilpiran et al., 2021).

### 2.4 MetS

Based on the National Cholesterol Education Program's Adult Treatment Panel (ATP) III, if each participant has three bottom criteria were considered as the MetS: SBP > 130 mmHg and/or DBP > 85 mmHg, fasting blood glucose (FPS) > 100 mg/dL, WC > 102 cm for men and > 88 cm for women, triglyceride (TG) ≥ 150 mg/dL, and high‐density lipoprotein‐cholesterol (HDL‐C) < 50 mg/dL for females and < 40 mg/dL for males (Askari et al., 2017).

### 2.5 Statistical analysis

All analyses were performed by SPSS version 23 software (IBM Corp.). The significance level of the tests was considered less than .05. At first, HBI was converted into quartiles, and then the analysis was performed accordingly. Mean and standard deviation (SD) or percentage were used to describe the basic differences between the HBI quartiles. The one‐way analysis of variance (ANOVA) or Kruskal–Wallis for quantitative and Chi‐square test for qualitative variables analysis were used. Univariate analysis was used to determine the relationship between HBI and MetS and its related factors. Multivariate analysis with the backward method was used to adjust potential confounders (age, body mass index (BMI), physical activity, education, energy intake, smoking, alcohol, medication use, and disease history).

## 3 RESULTS

Data from 8838 participants were included in the final analysis (Figure 1). There were significant differences in gender, smoking, alcohol history, medication, disease history, MetS prevalence, age, and physical activity among HBI quartiles (*p* ˂ .001 for all). Also, we observed a significant difference in BMI (*p* ˂ .001), WC (*p* ˂ .001), HC (*p* = .004), FBS (*p* = .006), total cholesterol (TC) (*p* = .005), and HBI score (*p* ˂ .001) among HBI quartiles. Moreover, individuals in the last quartile of HBI had a lower intake of energy and fat, but protein, carbohydrate, and fiber intakes were higher than the first quartile of HBI score (*p* ˂ .001 for all). But we observed no significant association among education, SBP, DBP, TG, and HDL‐C mean in HBI quartiles (*p* ˃ .05; Table 1).

**TABLE 1:** Baseline characteristics of study participants.

Variables	Healthy beverage index (*N* = 8838)					
	Q_1_ (*N* = 2210) (˂69)	Q_2_ (*N* = 2100) (69–73)	Q_3_ (*N* = 2766) (74–77)	Q_4_ (*N* = 1762) (≥78)	Total	*p*‐Value
Gender, male (%)	45.1	35.9	41.8	55.1	43.9	**˂.001**
Smoking, yes (%)	32.9	19.0	23.6	32.7	26.7	**˂.001**
Alcohol history, yes (%)	7.3	3.3	3.7	4.7	4.7	**˂.001**
Medication, yes (%)	25.7	14.3	17.8	25.3	20.5	**˂.001**
Disease history, yes (%)	55.1	61.4	60.5	61.2	59.5	**˂.001**
Metabolic syndrome (%)	21.1	20.1	21.4	15.8	19.9	**˂.001**
Age (year)	48.20 ± 9.65	48.02 ± 9.70	49.09 ± 9.46	49.45 ± 9.46	48.69 ± 9.58	**˂.001**
Education (year)	4.58 ± 3.88	4.81 ± 3.96	4.55 ± 3.77	4.69 ± 3.94	4.65 ± 3.88	.109
Physical activity (MET/day)	40.53 ± 11.19	40.59 ± 10.79	41.39 ± 10.71	43.24 ± 12.40	41.35 ± 11.25	**˂.001**
SBP (mmHg)	110.59 ± 18.11	111.81 ± 18.34	111.67 ± 18.36	111.23 ± 18.33	111.35 ± 18.29	.106
DBP (mmHg)	74.30 ± 11.85	75.04 ± 11.76	74.66 ± 11.73	74.36 ± 12.12	74.60 ± 11.85	.163
BMI (kg/m^2^)	25.63 ± 5.19	25.77 ± 4.78	25.90 ± 4.79	25.25 ± 4.58	25.67 ± 4.86	**˂.001**
WC (cm)	93.08 ± 12.51	93.35 ± 11.54	93.90 ± 11.71	92.23 ± 11.30	93.23 ± 11.81	**˂.001**
HC (cm)	99.60 ± 9.33	99.73 ± 8.68	100.01 ± 8.82	99.04 ± 8.39	99.65 ± 8.84	**.004**
FBS (mg/dL)	91.49 ± 27.06	91.77 ± 27.52	94.15 ± 31.25	92.60 ± 31.95	92.61 ± 29.55	**.006**
TG (mg/dL)	132.94 ± 77.55	131.11 ± 83.50	131.41 ± 79.30	129.11 ± 85.35	131.26 ± 81.12	.532
TC (mg/dL)	187.54 ± 40.45	185.30 ± 38.49	184.72 ± 38.03	183.23 ± 39.84	185.27 ± 39.14	**.005**
HDL‐C (mg/dL)	50.75 ± 15.91	51.65 ± 16.22	51.18 ± 15.98	50.79 ± 15.55	51.10 ± 15.93	.236
HBI Score	63.32 ± 4.11	71.68 ± 1.49	76.47 ± 0.92	80.92 ± 1.57	72.93 ± 6.75	**˂.001**
Energy (kcal/day)	2719.3 ± 780.6	2886.7 ± 691.1	2633.1 ± 665.6	2213.2 ± 666.7	2631.2 ± 738.4	**˂.001**
Protein (g/day)	95.04 ± 40.63	87.52 ± 34.61	91.78 ± 34.96	97.35 ± 41.99	82.69 ± 37.99	**˂.001**
Carbohydrate (g/day)	496.71 ± 202.79	442.15 ± 167.41	472.66 ± 172.43	519.52 ± 213.09	480.77 ± 189.81	**˂.001**
Fat (g/day)	73.07 ± 32.40	64.95 ± 26.55	68.32 ± 26.66	71.77 ± 29.66	69.40 ± 28.93	**˂.001**
Fiber (g/day)	31.37 ± 14.34	29.19 ± 12.09	30.78 ± 12.23	32.47 ± 14.19	30.89 ± 13.20	**˂.001**

*Note*: Values are mean (SD) for continuous and percentage for categorical variables. Using one‐way ANOVA for continuous and Chi‐square test for categorical variables in HBI tertiles.

Significant values are in bold.

Abbreviations: BMI, body mass index; DBP, diastolic blood pressure; FBS, fasting blood sugar; HBI, healthy beverage index; HC, hip circumference; HDL‐C, high‐density lipoprotein‐cholesterol; MET, metabolic equivalent of task; SBP, systolic blood pressure; TC, total cholesterol; TG, triglyceride; WC, waist circumference.

As shown in Table 2, individuals in the last quartile of HBI had significantly higher consumption of water, unsweetened coffee and tea, low‐ and full‐fat milk, and met fluid requirement (*p* ˂ .001 for all), also lower intake of SSBs (*p* < .001), but there were no differences in 100% fruit juice intakes among the HBI quartile (*p* ˃ .05).

**TABLE 2:** HBI components between quartiles of HBI.

Variables	Healthy beverage index (*N* = 8838)					
	Q_1_ (*N* = 2210) (˂69)	Q_2_ (*N* = 2100) (69–73)	Q_3_ (*N* = 2766) (74–77)	Q_4_ (*N* = 1762) (≥78)	Total	*p*‐Value
Water intake (mL/day)	450 (300–800)	610.7 (500–800)	800 (600–1050)	1014.2 (700–1300)	700 (500–1000)	**˂.001**
Unsweetened coffee and tea (mL/day)	690 (460–1150)	460 (230–690)	690 (460–1035)	920.2 (690–1725)	690 (460–1037.84)	**˂.001**
Low‐fat milk (mL/day)	47.89 (18.27–128.54)	47.89 (17.01–102.08)	65.53 (23.31–131.06)	65.53 (22.68–131.06)	55.45 (21.84–123.03)	**˂.001**
100% fruit juice (mL/day)	12.82 (4.93–33.20)	12.82 (4.84–30.06)	13.13 (5.91–32.05)	14.71 (4.97–34.19)	12.82 (5.32–32.05)	.135
Full‐fat milk (mL/day)	67.06 (32.93–117.30)	66.57 (32.93–115.08)	70.20 (33.89–130.72)	68.49 (33.56–131.06)	67.64 (33.26–118.16)	**˂.001**
Sugar‐sweetened beverages (mL/day)	105.86 (23.31–260.49)	34.15 (10.71–87.90)	37.80 (15.12–84.43)	42.47 (15.75–98.30)	46.31 (15.12–114.68)	**˂.001**
Total beverage (energy %)	1.0 (0.0–1.0)	1.0 (0.0–1.0)	0.0 (0.0–1.0)	1.0 (0.0–1.0)	1.0 (0.0–1.0)	**.012**
Met fluid requirement (mL/day)	1571.2 (1098.7–2251.4)	1318.1 (1053.4–1671.6)	1726.4 (1423.0–2105.1)	2264.1 (1779.8–2984.2)	1675.7 (1277.1–2231.4)	**˂.001**

*Note*: Values are median (Q1–Q3). Kruskal–Wallis test used for continuous variables in HBI tertiles.

Significant values are in bold.

Abbreviation: HBI, healthy beverage index.

According to Table 3, compared to the first quartile, we observed a significant association between the last quartile of the HBI with lower odds of MetS in univariate analysis (odds ratio [OR] = 0.70; 95% confidence interval [CI]: 0.52–0.82, *p* < .001). Also, compared to the first quartile, it was observed that HBI in the last quartile was significantly related to lower odds of MetS in the multivariate analysis (OR = 0.72; 95% CI: 0.60–0.87, *p* < .001).

**TABLE 3:** Association between study variables and metabolic syndrome in the total population of FASA cohort.

Variables	Metabolic syndrome			
	Total population (*N* = 8838)			
	Univariate OR (95% CI)	*p*‐Value	Multivariate OR (95% CI)	*p*‐Value
HBI score				
HBI Q_1_	Ref.	—	Ref.	—
HBI Q_2_	0.94 (0.81–1.09)	.438	0.86 (0.73–1.01)	.079
HBI Q_3_	1.02 (0.88–1.16)	.781	0.91 (0.78–1.06)	.239
HBI Q_4_	**0.70 (0.59–0.82)**	**˂.001**	**0.72 (0.60–0.87)**	**.001**
Demography				
Age (year)	**1.03 (1.03–1.04)**	**˂.001**	**1.03 (1.03–1.04)**	**˂.001**
BMI (kg/m^2^)	**1.20 (1.19–1.22)**	**˂.001**	**1.20 (1.17–1.20)**	**˂.001**
Physical activity (MET‐min/week)	**0.96 (0.96–0.97)**	**˂.001**	**0.98 (0.98–0.99)**	**˂.001**
Education (year)	**0.89 (0.88–0.91)**	**˂.001**	**0.98 (0.98–0.99)**	**˂.001**
Energy (kcal)	**1.00 (1.00–1.00)**	—	—	—
Smoke exposure				
No	Ref.	—	Ref.	—
Yes	**0.34 (0.29–0.39)**	**˂.001**	**0.55 (0.46–0.67)**	**˂.001**
Alcohol exposure				
No	Ref.	—	—	—
Yes	**0.33 (0.22–0.47)**	**˂.001**	—	—
Medication				
No	Ref.	—	Ref.	—
Yes	**0.29 (0.24–0.35)**	**˂.001**	**0.77 (0.62–0.95)**	**.020**
Disease history				
No	Ref.	—	Ref.	—
Yes	**2.90 (2.56–3.28)**	**˂.001**	**1.79 (1.57–2.05)**	**˂.001**

*Note*: Significant values are shown in bold. Missing values in each variable were excluded from the analyses. Using Backward LR method for multivariate analysis.

Abbreviations: BMI, body mass index; CI, confidence interval; HBI, healthy beverage index; MET, metabolic equivalent of task; OR, odds ratio.

The association between MetS components in univariate analysis is shown in Table 4. HBI in quartile 3 (Q_3_) was significantly related to higher odds of high FBS (OR = 1.24; 95% CI: 1.06–1.45, *p* = .006), but in Q_4_ was significantly associated with lower odds of high WC (OR = 0.66; 95% CI: 0.58–0.75, *p* < .001), high TG (OR = 0.84, 95% CI: 0.73–0.97, *p* = .020), and low HDL‐C (OR = 0.82; 95% CI: 0.72–0.93, *p* = .002). A multivariate analysis of MetS components is reported in Table 5. According to the results, a significant association was observed between the last quartile of HBI with lower odds of high WC (OR = 0.55; 95% CI: 0.45–0.67, *p* < .002).

**TABLE 4:** Association between study variables and metabolic syndrome components in the total population of FASA cohort in univariate analysis.

Metabolic syndrome components	High blood pressure		High fasting blood glucose		High waist circumference		High triglyceride		Low HDL‐C	
Variables	Univariate OR (95% CI)	*p*‐Value	Univariate OR (95% CI)	*p*‐Value	Univariate OR (95% CI)	*p*‐Value	Univariate OR (95% CI)	*p*‐Value	Univariate OR (95% CI)	*p*‐Value
HBI score										
HBI Q_1_	Ref.	—	Ref.	—	Ref.	—	Ref.	—	Ref.	—
HBI Q_2_	1.13 (0.97–1.32)	.098	1.01 (0.85–1.20)	.909	1.14 (1.01–1.29)	.027	0.96 (0.84–1.10)	.587	0.95 (0.84–1.08)	.482
HBI Q_3_	1.12 (0.97–1.29)	.116	**1.24 (1.06–1.45)**	**.006**	1.10 (0.98–1.23)	.083	0.97 (0.85–1.10)	.648	0.94 (0.84–1.05)	.303
HBI Q_4_	1.07 (0.91–1.26)	.361	0.97 (0.80–1.16)	.751	**0.66 (0.58–0.75)**	**˂.001**	**0.84 (0.73–0.97)**	**.020**	**0.82 (0.72–0.93)**	**.002**
Demography										
Age (year)	**1.06 (1.05–1.06)**	**˂.001**	**1.06 (1.06–1.07)**	**˂.001**	1.01 (1.00–1.01)	**˂.001**	**1.00 (1.00–1.01)**	**.005**	**0.98 (0.98–0.99)**	**˂.001**
BMI (kg/m^2^)	**1.09 (1.08–1.10)**	**˂.001**	**1.08 (1.06–1.09)**	**˂.001**	**1.56 (1.53–1.59)**	**˂.001**	**1.09 (1.08–1.10)**	**˂.001**	1.06 (1.05–1.07)	**˂.001**
Physical activity (MET‐min/week)	**0.98 (0.98–0.99)**	**˂.001**	**0.97 (0.96–0.98)**	**˂.001**	**0.95 (0.94–0.95)**	**˂.001**	**0.98 (0.98–0.99)**	**˂.001**	0.98 (0.97–0.98)	**˂.001**
Education (year)	**0.91 (0.91–0.93)**	**˂.001**	**0.88 (0.87–0.90)**	**˂.001**	**0.90 (0.89–0.91)**	**˂.001**	1.00 (0.98–1.01)	.921	0.99 (0.98–1.01)	.883
Energy (kcal)	1.00 (1.00–1.00)	.331	**1.00 (1.00–1.00)**	**.002**	**1.00 (1.00–1.00)**	**.013**	1.00 (1.00–1.00)	.078	1.00 (1.00–1.00)	.793
Smoke exposure										
No	Ref.	—	Ref.	—	Ref.	—	Ref.	—	Ref.	—
Yes	**0.71 (0.63–0.81)**	**˂.001**	**0.64 (0.56–0.74)**	**˂.001**	**0.13 (0.12–0.15)**	**˂.001**	0.95 (0.86–1.06)	.411	**0.79 (0.87–0.72)**	**˂.001**
Alcohol exposure										
No	Ref.	—	Ref.	—	Ref.	—	Ref.	—	Ref.	—
Yes	**0.53 (0.38–0.72)**	**˂.001**	**0.54 (0.38–0.76)**	**.001**	**0.12 (0.09–0.15)**	**˂.001**	1.01 (0.81–1.27)	.864	0.88 (0.72–1.08)	.238
Medication										
No	Ref.	—	Ref.	—	Ref.	—	Ref.	—	Ref.	—
Yes	**0.62 (0.53–0.72)**	**˂.001**	**0.62 (0.52–0.73)**	**˂.001**	**0.08 (0.08–0.10)**	**˂.001**	0.96 (0.86–1.08)	.588	**0.76 (0.69–0.85)**	**˂.001**
Disease history										
No	Ref.	—	Ref.	—	Ref.	—	Ref.	—	Ref.	—
Yes	**2.69 (2.38–3.04)**	**˂.001**	**3.33 (2.88–3.85)**	**˂.001**	**2.72 (2.49–2.97)**	**˂.001**	**1.31 (1.19–1.45)**	**˂.001**	**1.19 (1.09–1.30)**	**˂.001**

*Note*: Significant values are shown in bold. Missing values in each variable were excluded from the analyses. Using Backward LR method for multivariate analysis.

Abbreviations: BMI, body mass index; CI, confidence interval; HBI, healthy beverage index; HDL‐C, high‐density lipoprotein‐cholesterol; MET, metabolic equivalent of task; OR, odds ratio.

**TABLE 5:** Association between study variables and metabolic syndrome components in the total population of FASA cohort in multivariate analysis.

Metabolic syndrome components	High blood pressure		High fasting blood glucose		High waist circumference		High triglyceride		Low HDL‐C	
Variables	Multivariate OR (95% CI)	*p*‐Value	Multivariate OR (95% CI)	*p*‐Value	Multivariate OR (95% CI)	*p*‐Value	Multivariate OR (95% CI)	*p*‐Value	Multivariate OR (95% CI)	*p*‐Value
HBI score										
HBI Q_1_	—	—	Ref.	—	Ref.	—	—	—	—	—
HBI Q_2_	—	—	0.96 (0.80–1.16)	.700	0.95 (0.79–1.14)	.615	—	—	—	—
HBI Q_3_	—	—	1.16 (0.98–1.37)	.075	0.84 (0.70–1.00)	.052	—	—	—	—
HBI Q_4_	—	—	0.90 (0.74–1.10)	.341	**0.55 (0.45–0.67)**	**˂.001**	—	—	—	—
Demography										
Age (year)	**1.06 91.05–1.07)**	**˂.001**	**1.06 (1.05–1.07)**	**˂.001**	**0.99 (0.98–0.99)**	**.014**	**1.00 (1.00–1.01)**	**˂.001**	**0.98 (0.98–0.99)**	**˂.001**
BMI (kg/m^2^)	1.09 (1.07–1.10)	**˂.001**	**1.07 (1.05–1.08)**	**˂.001**	**1.64 (1.61–1.68)**	**˂.001**	**1.09 (1.08–1.10)**	**˂.001**	**1.05 (1.04–1.06)**	**˂.001**
Physical activity (MET‐min/week)	—	—	**0.98 (0.98–0.99)**	**˂.001**	**0.96 (0.95–0.96)**	**˂.001**	0.99 (0.99–1.00)	.090	**0.98 (0.98–0.98)**	**˂.001**
Education (year)	1.01 (0.99–1.03)	.085			**0.85 (0.83–0.86)**	**˂.001**	—	—	—	—
Energy (kcal)	—	—	1.00 (1.00–1.00)	.060	1.00 (1.00–1.00)	**.009**	**1.00 (1.00–1.00)**	**.037**	—	—
Smoke exposure										
No	Ref.	—	—	—	Ref.	—	—	—	—	—
Yes	**0.85 (0.74–0.98)**	**.031**	—	—	**0.25 (0.20–0.30)**	**˂.001**	—	—	—	—
Alcohol exposure										
No	—	—	—	—	Ref.	—	—	—	—	—
Yes	—	—	—	—	**0.63 (0.40–0.99)**	**.048**	—	—	—	—
Medication										
No	—	—	Ref.	—	Ref.	—	—	—	—	—
Yes	—	—	1.22 (0.99–1.49)	.058	**0.17 (0.13–0.22)**	**˂.001**	—	—	—	—
Disease history										
No	Ref.	—	Ref.	—	Ref.	—	—	—	—	—
Yes	**1.90 (1.67–2.17)**	**˂.001**	**2.29 (1.97–2.67)**	**˂.001**	**1.68 (1.47–1.92)**	**˂.001**	—	—	—	—

*Note*: Significant values are shown in bold. Missing values in each variable were excluded from the analyses. Using Backward LR method for multivariate analysis.

Abbreviations: BMI, body mass index; CI, confidence interval; HBI, healthy beverage index; HDL‐C, high‐density lipoprotein‐cholesterol; MET, metabolic equivalent of task; OR, odds ratio.

## 4 DISCUSSION

The present study demonstrated that Iranian adults with the highest HBI had significantly lower odds of MetS. Also, the findings indicated that those with the highest HBI were likely to have lower WC than those with the lowest HBI after adjusting for confounders. Regarding other components of MetS, no significant relationship was found with HBI scores in the adjusted model. Further, the results showed that in a higher HBI, intake of water, unsweetened coffee and tea, low‐fat milk, and full‐fat milk was significantly higher than in a lower HBI. In contrast, SSB intake was significantly lower in the last quartile of HBI compared to the first quartile.

The HBI was applied to assess the overall quality of beverage intake and determine whether changes in beverage consumption patterns were related to improvements in a health condition (Duffey & Davy, 2015). The HBI components include total beverage energy, fluid intake, and eight categories of beverages (Hedrick et al., 2015). As shown in the present study, water was the most important source of overall fluid intake, and people located in the highest tertile of HBI retained more fluid intake and met the need for fluids.

The current study showed an inverse association between HBI and MetS. A study by Liu et al. (2021) revealed that a higher HBI is negatively associated with MetS in females in the United States (US). Also, Shin et al. (2018) reported that SSB consumption was positively related to MetS odds (OR: 1.61; 95% CI: 1.20–2.16; *p* for trend = .0003). Moreover, another study on Mexican adults showed that sweetened beverage intake increases MetS risk (Denova‐Gutiérrez et al., 2010). Studies have shown that the incidence of MetS was 0.5 to 2 times higher in people who consumed more SSBs (Barrio‐Lopez et al., 2013; Dhingra et al., 2007). A study by Mirmiran et al. (2015) showed that sugar‐sweetened carbonated soft drinks were positively associated with MetS, hypertension, and abdominal obesity and greater odds of adverse changes in cardiometabolic risk factors, independent of body weight. Mechanisms explaining the association between SSBs and MetS include that liquid carbohydrates induce less satiety than solid carbohydrates (Cassady et al., 2012; Pan & Hu, 2011); thus, they increase energy intake and weight gain (Vartanian et al., 2007). Also, SSB consumption causes insulin resistance (Mirmiran et al., 2015).

A negative association was observed between HBI and WC. A study by Duffey and Davy (2015) demonstrated that in higher HBI, the odds of having a high WC decrease. A study by Collison et al. (2010) showed that higher sugar‐sweetened carbonated beverages were associated with higher WC in school boys. Moreover, a study conducted by Funtikova et al. (2015) on Spanish adults proved that replacing soft drinks containing 100 kcal with fruit juice and whole milk holding 100 kcal reduces WC by 1.1 and 1.3 cm, respectively. Further, a meta‐analysis of a prospective cohort study by Ardeshirlarijani et al. (2021) showed a negative association between SSB consumption and WC. SSB can increase WC for the following reasons: They can stimulate higher glycemic foods consumption and increase calorie intake. Also, more insulin is needed due to the high glycemic load, and the increase in insulin secretion causes fat accumulation and weight gain (Papier et al., 2017).

An inverse relationship was detected between HBI and high FBS, but it was insignificant. Our finding is consistent with a similar study. A study on US adults found an inverse association between HBI and high FBS in women, which was insignificant (Duffey & Davy, 2015).

There were limitations to the current study. First, due to its cross‐sectional nature, causality could not be confirmed. Second, there might be confounding factors that were not considered in the study. Third, using FFQ, it is impossible to estimate the participants' real intake. However, the FFQ is an easy method to collect dietary information in epidemiological studies. Also, the present study had some strengths. Data were extracted from the FASA cohort with a large sample. Moreover, the relationship between MetS components and HBI was evaluated. In addition, collecting demographic and social information on the study population facilitated a comprehensive examination of possible confounding factors. Consequently, several significant confounding variables were adjusted for all analyses.

## 5 CONCLUSIONS

Our findings showed that adherence to a healthy beverage pattern is associated with lower odds of developing MetS and high WC. Therefore, to reduce the odds of MetS, a healthy pattern of beverage consumption, including drinking water, low‐fat milk, unsweetened tea, and coffee, and reducing the consumption of SSB are recommended. More studies are needed to confirm the findings.

## AUTHOR CONTRIBUTIONS

**Mehran Nouri:** Formal analysis (equal); investigation (equal); writing – original draft (equal). **Zainab Shateri:** Writing – original draft (equal). **Mahshid Rezaei:** Writing – original draft (equal). **Ali Zangene:** Writing – original draft (equal). **Reza Homayounfar:** Data curation (equal); methodology (equal); writing – review and editing (equal). **Parisa Keshani:** Investigation (equal); methodology (equal); writing – review and editing (equal).

## CONFLICT OF INTEREST STATEMENT

The authors declare that they have no conflict of interest.

## Data Availability

The data that support the findings of this study are available from the corresponding author upon reasonable request.
